# Self‐Healable, Self‐Repairable, and Recyclable Electrically Responsive Artificial Muscles

**DOI:** 10.1002/advs.202202153

**Published:** 2022-06-03

**Authors:** Johannes von Szczepanski, Patrick M. Danner, Dorina M. Opris

**Affiliations:** ^1^ Laboratory for Functional Polymers Swiss Federal Laboratories for Materials Science and Technology Empa Ueberlandstr. 129 Dübendorf 8600 Switzerland; ^2^ Department of Materials ETH Zurich Vladimir‐Prelog‐Weg 5 Zurich 8093 Switzerland

**Keywords:** dielectric elastomer actuators, electrically responsive polymers, high‐permittivity elastomers, recycling, self‐healing, soft actuators, soft robotics

## Abstract

Elastomers with high dielectric permittivity that self‐heal after electric breakdown and mechanical damage are important in the emerging field of artificial muscles. Here, a one‐step process toward self‐healable, silicone‐based elastomers with large and tunable permittivity is reported. Anionic ring‐opening polymerization of cyanopropyl‐substituted cyclic siloxanes yields elastomers with polar side chains. The equilibrated product is composed of networks, linear chains, and cyclic compounds. The ratio between the components varies with temperature and allows realizing materials with largely different properties. The silanolate end groups remain active, which is the key to self‐healing. Elastomeric behavior is observed at room temperature, while viscous flow dominates at higher temperatures (typically 80 °C). The elasticity is essential for reversible actuation and the thermoreversible softening allows for self‐healing and recycling. The dielectric permittivity can be increased to a maximum value of 18.1 by varying the polar group content. Single‐layer actuators show 3.8% lateral actuation at 5.2 V µm^–1^ and self‐repair after a breakdown, while damaged ones can be recycled integrally. Stack actuators reach an actuation strain of 5.4 ± 0.2% at electric fields as low as 3.2 V µm^–1^ and are therefore promising for applications as artificial muscles in soft robotics.

## Graphical abstract

A straightforward synthesis of elastomers with tunable dielectric permittivity and mechanical properties is presented. The elastomers self‐heal when damaged, can be thermoplastically processed, and recycled. The materials are used to fabricate actuators that self‐repair after a breakdown and regain the previous performance. Stack actuators can be manufactured easily and reach 5.4% strain at a low electric field of 3.2 V µm^–1^.

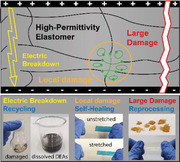

## 1 Introduction

The demand for new self‐healable and stimuli‐responsive functional polymers increased significantly during the last few years.^[^ ^1^, ^2^ ^]^ Self‐healing ability can be introduced extrinsically by blending the material with healing agents released during damage or intrinsically by reversible bonds that break and reform after damage.^[^ ^3^ ^]^ Extrinsic self‐healing is time‐dependent since the available amount of healing agents decreases over time. Contrary, intrinsic self‐healing allows for repetitive healing by external stimuli such as temperature or light with preservation of key properties.^[^ ^3^ ^]^ Reversible covalent bonds such as Diels–Alder reactions^[^ ^4^, ^5^ ^]^ and interconversion between disulfide groups and thiols^[^ ^6^, ^7^, ^8^, ^9^ ^]^ as well as noncovalently bonded systems such as hydrogen bonds,^[^ ^10^ ^]^ *π*–*π* stacking,^[^ ^11^, ^12^, ^13^ ^]^ metal‐ligand complexes,^[^ ^14^, ^15^ ^]^ ionic,^[^ ^16^ ^]^ and host‐guest interactions have been used.^[^ ^17^ ^]^

The anionic equilibration of cross‐linked polydimethylsiloxane (PDMS) was recently reintroduced by McCarthy et al. as a fast and efficient self‐healing method.^[^ ^18^ ^]^ The silanolate chain ends remain in a “living” state in this reaction. Consequently, the equilibrium between cross‐linked networks and cyclic compounds can be shifted as a function of temperature. Still, these materials need to be heated to 90 °C for 24 h to self‐heal. Seiffert et al. achieved room temperature self‐healable PDMS by increasing the number of anionic groups in the network.^[^ ^19^ ^]^ These reversible PDMS networks lose the ability to self‐heal when heated above 150 °C, as the active chain ends are decomposed.^[^ ^20^ ^]^

Despite the abundant research on self‐healable materials, reports on healable elastomers with high dielectric permittivity remain limited. Such materials are of great interest for many applications, including stretchable electronics, lithium‐ion batteries, and dielectric elastomer actuators (DEAs).^[^ ^21^ ^]^ DEAs are a class of artificial muscles that can convert an electrical stimulus into mechanical work.^[^ ^22^, ^23^, ^24^ ^]^ They are characterized by outstanding shape flexibility and elasticity compared to commonly used rigid transducers.^[^ ^23^, ^25^, ^26^ ^]^ The term artificial muscles is used to describe a broad number of different stimuli‐responsive soft actuators, including pneumatic actuators,^[^ ^27^ ^]^ shape memory alloys,^[^ ^28^ ^]^ temperature‐responsive hydrogels,^[^ ^29^ ^]^ and electroactive polymers.^[^ ^30^ ^]^ The advantages of DEAs compared to other stimuli‐responsive systems include large strains, fast response, and the easily controllable electrical stimulus.^[^ ^31^ ^]^ Just like natural muscles, DEAs are exposed to the risk of damage (e.g., by a dielectric breakdown or mechanical rupture).^[^ ^32^ ^]^ Large electric fields with voltages in the kV range, even for thin films, need to be applied across the electrodes for actuation, which increases the probability of a dielectric breakdown event. In this case, the electrodes suddenly discharge and a high current flows through the elastomer. The generated heat can cause burning of the dielectric membrane and failure of the device.^[^ ^33^ ^]^ Whenever the durability of a device is of concern, it is attractive and sustainable if the employed material can self‐heal.^[^ ^34^ ^]^ So far, only a few examples of intrinsically self‐healing dielectric elastomers are known. Madsen et al. achieved a self‐healable interpenetrating polymer network by using two different networks formed by chemical and ionic bonds. These elastomers possess a permittivity of up to 6.3 at 0.1 Hz, but have not been tested as dielectrics in actuators.^[^ ^35^ ^]^ Li and co‐workers used metal‐ligand coordination to achieve a self‐healable dielectric elastomer. Their material has a permittivity of 6.4 at 10 kHz and an actuation strain of 3.6% at an electric field of 17.2 V µm^–1^, which was reached at 11 kV.^[^ ^36^ ^]^ Wan et al. developed styrene–butadiene–styrene elastomers modified with thioglycolate side groups that reach a permittivity of 11.4 at 1 kHz.^[^ ^37^, ^38^, ^39^ ^]^ The actuators gave a lateral strain of 4.5% at a high electric field of 17.6 V µm^–1^.^[^ ^39^ ^]^ Liu et al. developed a composite of TiO_2_‐urea core–shell particles in polyurea,^[^ ^40^ ^]^ which showed a permittivity of 16.1 at 1 kHz and a lateral actuation of 7.5% at an electric field of 8.5 V µm^–1^.^[^ ^40^ ^]^ Duan et al. synthesized a polysiloxane elastomer that self‐heals through hydrogen bonds between carboxylic acid and polyaniline side groups and reached a permittivity of 11.1 at 50 kHz.^[^ ^41^ ^]^ After a dielectric breakdown, the actuation decreased from 7% strain for the initial actuator to 1.62% strain for the self‐healed one at an electric field of 15.8 V µm^–1^.^[^ ^41^ ^]^

Although self‐healing increases the actuators' reliability and lifetime, they can still become irreversibly damaged. In this case, recycling would allow the re‐use of the base materials and reduce the environmental impact. However, to the best of our knowledge, recyclability has never been shown for any dielectric elastomer material. Recycling is especially important in manufacturing stack devices, where about 20–30% fail the reliability tests,^[^ ^42^ ^]^ and in single‐use applications, such as disposable patches for health monitoring.^[^ ^43^ ^]^

In this work, we developed a simple, one‐step synthesis of high‐permittivity dielectric elastomers that can self‐heal after damage and be used to fabricate recyclable actuators. We synthesized the elastomer by anionic ring‐opening polymerization of 1,3,5,7‐tetramethyl‐1,3,5,7‐tetra(3‐cyanopropyl)cyclotetrasiloxane (D_4_^CN^), octamethylcyclotetrasiloxane (D_4_), and a specially designed *co*‐monomer (tris‐D_4_) that functions as cross‐linker. After equilibration, a “living” network is formed, where the active silanolate end groups are responsible for the self‐healing ability. The dielectric properties can be easily tuned by varying the ratio of polar and nonpolar monomers and the mechanical properties can be optimized by changing the amount of tris‐D_4_ cross‐linker. The formed elastomers self‐heal through reversible covalent bonds. Additionally, the material can be processed into thin films by melt pressing, and single membrane and stack actuators can be manufactured easily. The actuators respond at low electric fields and self‐repair after damage to function with unaltered performance. Furthermore, we demonstrate that the actuators can be recycled in a simple and scalable way.

## 2 Synthesis and Characterization

**Scheme 1:**
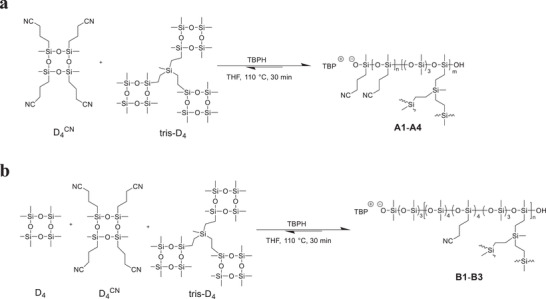
a) Synthesis of thermoreversible, high‐permittivity elastomers with 100% polar groups (**A1**‐**A4**) by anionic ring‐opening polymerization (AROP) starting from cyclic monomer D_4_^CN^ and trifunctional cross‐linker tris‐D_4_. b) Synthesis of elastomers with 50% polar groups (**B1**‐**B3**) by substituting half of the amount of polar monomer D_4_^CN^ with nonpolar D_4_.

Siloxane equilibration was recently re‐discovered as an efficient and straightforward self‐healing mechanism. Polysiloxanes prepared by anionic ring‐opening polymerization (AROP) can undergo rearrangement of covalent bonds at elevated temperatures.^[^ ^18^, ^19^ ^]^ The system reaches an equilibrium state between a cross‐linked network and low molar mass cyclic compounds. Based on this approach, we synthesized thermoreversible elastomers by polymerizing cyclic siloxanes at elevated temperatures and subsequent cross‐linking upon cooling to room temperature. We combined a polar monomer (D_4_^CN^) with a nonpolar trifunctional cross‐linker (tris‐D_4_). Both were synthesized by hydrosilylation reaction according to modified literature procedures (Figures S1–S3, Supporting Information).^[^ ^20^, ^44^ ^]^ AROP of monomer D_4_^CN^ and tris‐D_4_ cross‐linker with tetrabutylphosphonium hydroxide (TBPH) initiator yields elastomers **A1**‐**A4** (**Scheme** **1**a). The elastic networks consist of a) linear segments with varying cyanopropyl group content that can be easily tuned by varying the monomer feed ratio, b) cross‐links that are introduced by the trifunctional cross‐linker and whose density can be tuned by changing the amount of cross‐linker used, and c) active silanolate chain ends. These silanolate end groups provide the self‐healing mechanism, as they can initiate chain cleavage through backbiting at increased temperatures. The material reversibly softens upon heating and self‐heals after damage. The cyanopropyl groups increase the reactivity of the cyclic siloxane compared to the nonpolar D_4_, which results in an increased depolymerization rate at elevated temperatures. Hence, the polar groups allow for faster self‐healing than previously reported PDMS‐based systems, as well as full recyclability.

Four elastomers, **A1**‐**A4** were synthesized with different concentrations of tris‐D_4_ cross‐linker, which allowed for tuning the mechanical properties (**Table** **1**). Tensile tests reveal a decrease of elongation at break with increasing cross‐linker concentration from 59 ± 3% for elastomer **A1** to 41 ± 3% for elastomer **A4** (Table 1, Figure S4, Supporting Information). At the same time, the Young's modulus rises with increasing concentration of tris‐D_4_ from 130 ± 4 kPa up to 202 ± 10 kPa. To improve the strain at break and reduce viscoelastic losses at high frequencies, we also synthesized three elastomers with lower content of polar cyanopropyl groups (**B1**‐**B3**). Therefore, we substituted 50% of monomer D_4_^CN^ with nonpolar monomer octamethylcyclotetrasiloxane (D_4_) (**Scheme** **1**b). The better compatibility between nonpolar cross‐linker tris‐D_4_ and nonpolar monomer D_4_ leads to a more effective network formation. Consequently, the elastomers with 50% polar groups show a higher Young's modulus at the same cross‐linker concentration than those with 100% polar groups. The elongation at break is larger for the elastomers **B1** and **B2** than for the homopolymers **A1** and **A2**, due to the higher flexibility of the dimethylsiloxane units. This effect is outweighed by the more effective network formation for elastomer **B3**, for which the elongation at break decreases to 33 ± 1% compared to 43 ± 2% for elastomer **A3**.

**Table 1:** Amount of reagents used to synthesize elastomers **A1**‐**A4** and **B1**‐**B3** and key properties of the resulting materials including *T*_g_, Young's modulus at 10% strain (*Y*_10%_), elongation at break, and permittivity at 100 kHz (*ε*'). The amount of initiator tetrabutylphosphonium hydroxide (TBPH) was kept constant at 25 µL (0.92 mol%)

Elastomer	D_4_^CN^ [g]	D_4_^CN^ [mol%]	D_4_ [g]	D_4_ [mol%]	tris‐D_4_ [mg]	tris‐D_4_ [mol%]	*T*_g_ [°C]	*Y*_10%_ [kPa]^a)^	Elong. [%]	*ε'* (100 kHz)
**A1**	2	100	–	–	20	0.52	−59.0	130 ± 4	59 ± 3	18.1 ± 0.1
**A2**	2	100	–	–	50	1.3	−59.1	168 ± 12	48 ± 1	17.4 ± 0.6
**A3**	2	100	–	–	100	2.6	−59.0	176 ± 6	43 ± 2	17.3 ± 0.2
**A4**	2	100	–	–	150	3.9	−58.8	202 ± 10	41 ± 3	16.9 ± 0.3
**B1**	1	50	0.59	50	20	0.52	−74.3	178 ± 8	87 ± 6	12.7 ± 0.7
**B2**	1	50	0.59	50	50	1.3	−73.8	274 ± 3	64 ± 5	12.7 ± 0.2
**B3**	1	50	0.59	50	100	2.6	−74.1	444 ± 15	33 ± 1	12.7 ± 0.3

^a)^ Young's moduli were determined from the slope of the stress–strain curves with a linear fit in the first 10% of strain.

**Figure 1:**
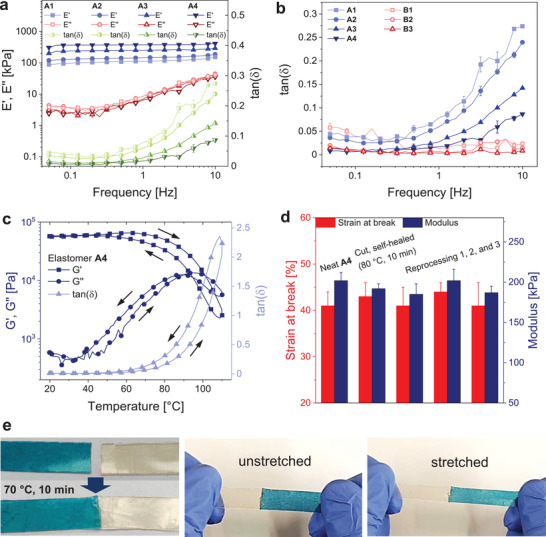
a) DMA traces of elastomers **A1**‐**A4**. The Young's modulus increases with increasing cross‐linker concentration; at the same time, viscous losses decrease. If not shown, error bars are smaller than the symbol size. b) tan(*δ*) of elastomers **A1**‐**A4** and **B1**‐**B3**, elastomers with 50% polar groups show lower viscous losses at high frequencies compared to elastomers with 100% polar groups. If not shown, error bars are smaller than the symbol size. c) Thermoreversible properties of elastomer **A4** are proven by temperature‐dependent shear rheology measurements (heating and cooling rate: 3 K min^–1^, angular frequency: 1 rad s^–1^, strain: 0.1%). d) Self‐healing experiments of elastomer **A4**. The initial mechanical properties are restored after cutting and heating to 80 °C for 10 min. Also, by up to three processing cycles the mechanical properties are only slightly impaired. e) Two stripes of elastomer **B1** are joined by self‐healing at 70 °C for 10 min and can be stretched without rupturing at the connecting site. The addition of soluble phthalocyanine colorized the stripe in blue.^[^ ^46^ ^]^

DMA measurements confirm the trends for the mechanical properties. For all samples, the storage modulus increases with increasing cross‐linker concentration (**Figure** **1**a and Figure S5, Supporting Information). Elastomers **A1**‐**A4** show higher viscous losses, when decreasing the concentration of tris‐D_4_, which can be observed as an increase of tan(*δ*) at frequencies above 0.5 Hz (Figure 1b). On the contrary, the tan(*δ*) of elastomers **B1**‐**B3** takes very small values over the whole frequency range (0.05–10 Hz) and a trend toward higher viscous losses is not observed. Again, this is a consequence of the more effective network formation, ensuring high flexibility even at low cross‐linker concentrations.

We conducted temperature‐dependent rheology measurements of elastomers **A4** and **B1** to prove the thermoreversible nature of the polymer network (Figure 1c and Figure S6, Supporting Information). The storage modulus G′ at room temperature outweighs the loss modulus *G″* by far. Upon heating, the network is partially cleaved, the material becomes gradually softer, and the viscosity decreases (Figure S6, Supporting Information). At 110 °C, *G″* has increased above *G′* and the material is in a highly viscous fluid state. When cooling back to room temperature, the network is re‐formed and the initial mechanical properties are restored. This process of network re‐formation during cooling is slower than the network cleavage upon heating, due to decreased mobility of the active chain ends when cooling. Thus, the storage modulus and the viscosity show a small hysteresis. All characterizations of the elastomers were conducted at least 24 h after processing.

The thermoreversible nature of the elastomer system opens new possibilities for the processing into thin films and the fabrication of DEAs in a continuous, extrusion‐based process.^[^ ^45^ ^]^ We prepared elastomer thin films by melt pressing at temperatures between 60 °C and 100 °C. We investigated the self‐healing capability and recyclability of the elastomer system by repeated tensile tests exemplified for elastomer **A4**. Tensile test specimens were melt pressed at 80 °C for 1 min with a pressure of 1 t after 5 min equilibration at 80 °C. For a self‐healing experiment, the samples were cut with a blade. By heating to 80 °C for 10 min, the initial mechanical properties could be restored (Figure 1d). At 80 °C, the material is partially depolymerized and becomes viscous‐like (Figure 1c). The increased polymer chain mobility facilitates the self‐healing process. Applying a constant load during the self‐healing process would lead to an irreversible deformation of the material as the viscous properties are predominant. Therefore, self‐healing can only take place while the material is in a relaxed state. We could also completely reshape the elastomer to new test specimens by melt‐pressing three times while the mechanical properties were only slightly impaired (Figure 1d). Similar self‐healing and reprocessing experiments were carried out for elastomer **A2** (Figure S7, Supporting Information). Due to the lower cross‐linker concentration in elastomer **A2**, a lower self‐healing temperature of 70 °C was sufficient to restore the initial mechanical properties. To visualize the self‐healing process, we connected two stripes of elastomer **B1** (Figure 1e). Also here, a temperature of 70 °C for 10 min was sufficient for the self‐healing process. The resulting film could be stretched without rupturing at the connecting site.

**Figure 2:**
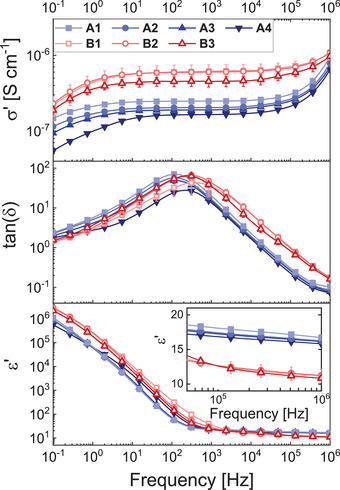
Conductivity (*σ*'), dielectric loss tangent (tan(*δ*)), and permittivity (*ε*') of the seven different elastomers measured at RT as a function of frequency.

Dielectric impedance spectroscopy provides information about the dielectric properties of the elastomers. The results for conductivity (*σ'*), dielectric loss tangent (tan (*δ*)), and relative permittivity (*ε'*) are plotted in **Figure** **2** as a function of frequency. All elastomers exhibit quite large values for the conductivity of up to 10^–6^ S cm^–1^, likely caused by the ionic initiator TBPH. The conductivity decreases with increasing cross‐linking density, as the ionic mobility is progressively impaired. The elastomers with 50% polar groups show an increased conductivity, likely due to a higher weight percentage of initiator TBPH and increased mobility of the polymer chains due to the lower *T*_g_. The phase angle, *θ*, between voltage and current is plotted in Figure S8 (Supporting Information) for the elastomers **A4** and **B1**. The materials show a capacitive behavior at high frequencies (*θ* = 90°) and a conductive behavior in the frequency range of 100–1000 Hz (*θ* = 0°). The conductive behavior is also indicated by a frequency‐independent, constant conductivity regime in Figure 2. At low frequencies, the material behaves again more capacitive. The large conductivity makes the material unsuitable for energy harvesting applications. However, actuator and sensor applications are attractive, as they don't require very high voltages, if the film thickness is sufficiently reduced. The drawback of high conductivity could be overcome in future work by coating the elastomer membrane with a very thin PDMS blocking layer.

The permittivity shows a linear increase toward low frequencies due to electrode polarization. Here, mobile ionic residues accumulate at the electrodes leading to interfacial polarization. At high frequencies, the alignment of the polar side groups in the electric field determines the permittivity. Elastomers **A1**‐**A4** exhibit high permittivity values between 16.9 ± 0.3 and 18.1 ± 0.1 at a frequency of 100 kHz (Table 1). The permittivity decreases slightly with increasing content of nonpolar cross‐linker tris‐D_4_ from **A1** to **A4**. The mean value of 17.6 is in good accordance with literature values of nitrile group modified polysiloxanes of 17.4.^[^ ^47^ ^]^ For elastomers **B1**‐**B3** a lower permittivity of 12.7 is measured, due to dilution of the cyanopropyl dipoles. Elastomers **A1**‐**A4** have a permittivity value of less than twice the permittivity of the elastomers with 50% polar groups. The increasing number of dipoles impairs the mobility of the chain segments and the alignment of the dipoles in the electric field. The decreased mobility can also be observed by a shift of the peak of tan(*δ*) toward slightly lower frequencies for the 100% polar groups polymers. This effect also increases the *T_g_* from −74 °C for elastomers **B1**‐**B3** to −59 °C for elastomers **A1**‐**A4** (Table 1, Figure S9, Supporting Information). A similar observation was also reported in previous studies.^[^ ^48^ ^]^

## 3 Actuator Performance, Self‐Repairing, and Recycling

**Figure 3:**
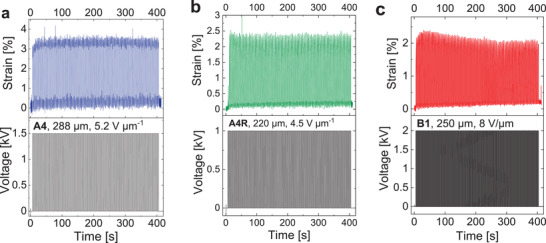
Cyclic testing of three single‐layer actuators prepared from elastomers **A4**, **B1**, and **A4R** at a frequency of 0.25 Hz. a) Membrane of elastomer **A4** with a thickness of 288 µm operated at 5.2 V µm^–1^ (1500 V). b) Membrane of recycled elastomer **A4R** with a thickness of 220 µm operated at 4.5 V µm^–1^ (1000 V). c) Membrane of elastomer **B1** with a thickness of 250 µm operated at 8.0 V µm^–1^ (2000 V).

We prepared elastomer thin films for the actuator fabrication by melt pressing. After pre‐stretching by 14.3%, the thickness of the membranes was in the range of 200–300 µm. Carbon black was applied on both sides of the membrane to serve as circular electrodes. During the actuation cycles, a camera measures the relative expansion of the carbon black electrodes. The tendency for viscous losses increases with decreasing cross‐linker concentration from elastomer **A4** to **A1** (Figure 1b). Viscous losses result in an increase of the strain baseline after repeated actuation. Therefore, the actuators prepared from elastomer **A4** with the lowest viscous losses performed best. They could be repeatedly cycled at high frequencies and showed no change over time. One device reached a lateral strain of 3.8% at a low electric field of 5.2 V µm^–1^ (**Figure** **3**a). The actuation is stable over 100 cycles without viscous losses. Also, the actuator can self‐repair after an electric breakdown, as shown in Figure S10 (Supporting Information). Here, the actuator was operated very close to the electric breakdown. After the first breakdown event in cycle 16, the actuator returns to the initial actuation in the following cycle. Only after repeated, more severe breakdowns, the actuation could not be recovered completely. This self‐repairing effect is most likely caused by self‐clearing of the actuator membrane. In this case, the heat generated during breakdown leads to local burning of dielectric and electrode. Consequently, the burned area is deactivated and becomes electrically insulating.^[^ ^49^ ^]^ This behavior has previously been reported by our group for actuators prepared from polysiloxanes with nitrile side groups.^[^ ^50^ ^]^ As more and more parts of the membrane area are deactivated, the actuation performance decreases after repeated breakdown events.^[^ ^49^ ^]^

The described self‐clearing effect can increase the lifetime of devices but does not remove the defect in the structure of the dielectric and electrode. In the case of accumulated local defects or large‐scale damages, recycling the device is the most sustainable option. We tested the recycling of actuator membranes for elastomer **A4**. At elevated temperatures, the network is partially cleaved and the equilibrium is shifted to the side of low molar mass compounds. Therefore, the process is based on the same molecular approach as the self‐healing mechanism. The depolymerization was triggered by heating used actuator membranes prepared from elastomer **A4** in THF to 80 °C under reflux. THF promotes backbiting of the active silanolate end groups and therefore shifts the equilibrium toward low molar mass cyclic siloxanes. Afterward, we removed the carbon black powder of the electrodes by filtration, followed by solvent removal. Polymerization of the low molar mass residue at 110 °C yields a new dielectric elastomer (**A4R**). The recycled material **A4R** shows similar mechanical properties to the neat elastomer **A4** in tensile tests and DMA, as well as a similar *T*_g_ (Figures S4, S5, and S9, Supporting Information). TGA reveals that the thermal stability of the recycled material is slightly impaired (Figure S9, Supporting Information). A new actuator prepared from elastomer **A4R** reaches a lateral actuation strain of 2.5% at an electric field of 4.5 V µm^–1^ (Figure 3b). As the actuation strain depends on the square of the electric field, the relative actuation strain of 2.5% at 4.5 V µm^–1^ is only slightly lower than the strain of 3.8% at 5.2 V µm^–1^ observed for the neat elastomer **A4**. The recycling strategy can be applied equally to the materials **B1**‐**B3** with 50% polar groups based on the same molecular mechanism. The elastomers with 50% polar groups (**B1**‐**B3**) do not suffer from viscous losses (Figure 1b). Between these three materials, elastomer **B1** benefits from a low Young's modulus. Therefore, we observed the best performance for an actuator prepared from **B1**. In cycling testing, the device reached a strain of 2.4% at an electric field of 8.0 V µm^–1^ (Figure 3c). One actuator even reached a strain of 12.3% at an electric field of 7.2 V µm^–1^ in a step increase experiment (Figure S11, Supporting Information).

**Figure 4:**
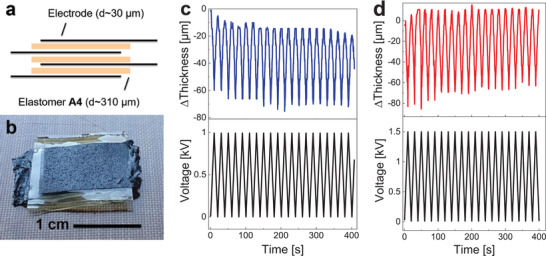
a) Stack actuator design. b) Stack actuator prepared from three active layers of elastomer **A4** and four electrode layers made from polydimethylsiloxane (PDMS) blended with graphite nanoplatelets. c) Performance of a stack actuator with three active layers of elastomer **A4** (310 µm each) and four layers of electrode (30 µm each). At an electric field of 3.2 V µm^–1^ a thickness change of 56 ± 2 µm is observed, corresponding to a relative actuation of 5.4 ± 0.2%. d) Stack actuator with five active layers of elastomer **B1** (225 µm each) and six layers of electrode (62 µm each). At an electric field of 6.7 V µm^–1^ a thickness change of 80 ± 6 µm is observed, corresponding to a relative actuation of 4.9 ± 0.4%. Both actuators were operated at 25 mHz.

Previous work on high‐permittivity elastomers mostly focused on the synthesis and characterization of the material as well as testing of single‐layer actuators. The next step toward applying the materials is the fabrication of simple soft robotic devices. One class of simple devices is stack actuators that are obtained by placing several single‐layer actuators on top of each other.^[^ ^45^, ^51^, ^52^ ^]^ We prepared stack actuators of elastomers **A4** and **B1** by manually stacking alternating layers of dielectric elastomer and electrode (**Figure** **4**a).

As electrode material, we used a film of cross‐linked PDMS blended with graphite nanoplatelets.^[^ ^46^ ^]^ The stack of elastomer **A4** consists of three active layers of dielectric elastomer and four layers of electrode (Figure 4b). We tested the actuator performance at a voltage of 1000 V with a frequency of 25 mHz (Figure 4c). When a voltage is applied, the electrostatic pressure of the electrodes leads to a compression of the elastomer films in the cross‐plane direction. Consequently, the thickness of the stack decreases. When the voltage is turned off, the actuator relaxes back to the initial position (Video S1, Supporting Information). The actuator shows an average thickness change of 56 ± 2 µm corresponding to an actuation of 5.4 ± 0.2% at a very low electric field of 3.2 V µm^–1^ (**Table** **2**). The baseline increase can be attributed to the viscoelastic losses of the dielectric elastomer. The actuator prepared from elastomer **B1** consists of five active dielectric elastomer layers and six electrode layers. We observed an average change in stack thickness of 80 ± 6 µm and an average actuation of 4.9 ± 0.4% at 6.7 V µm^–1^ (Figure 4d and Video S2, Supporting Information). The actuation strain decreases after several cycles. To the best of our knowledge, these actuators are the first stack actuators with high‐permittivity polysiloxanes as dielectric ever reported.^[^ ^47^ ^]^

**Table 2:** Key performance parameters for the two stack actuators prepared from elastomers **A4** and **B1**. The electrostatic pressure, *p*, and the generated force, *F*, were calculated according to Equations (1) and (2)

Stack	Elastomer	Layer thickness [µm]	*V* [kV]	*E* [V µm^–1^]	*s*_z_ [%]	*p* [kPa]	*n*	*A* [cm^2^]	*F* [N]
1	**A4**	310	1.0	3.2	5.4 ± 0.2	1.56	3	1.0	0.47
2	**B1**	225	1.5	6.7	4.9 ± 0.4	5.00	5	1.0	2.50

The electrostatic pressure, *p*, and the force, *F*, generated by the stack actuators can be calculated according to Equations (1) and (2).^[^ ^53^ ^]^

(1) $$p=\varepsilon_{0}\varepsilon_{r}E^{2}$$

(2) F=npA

Here, *ε_0_ =* 8.854 × 10^‐12^ A s V^–1^ m^–1^ is the vacuum permittivity, *ε_r_* is the relative permittivity of the dielectric elastomer, *E* is the electric field strength, *n* is the number of active layers in the stack, and *A* is the active area of each layer. We calculated the electrostatic pressure and the generated force for both stack actuators given the electric field applied during the measurement shown in Figure 4. Stack 1 prepared from elastomer **A4** reaches a pressure of 1.56 kPa, corresponding to a force of 0.47 N for the active area of 1 cm^2^ and three active layers (Table 2). Stack 2 prepared from elastomer **B1** gives a larger electrostatic pressure of 5.00 kPa due to the higher operating field of 6.7 V µm^–1^. With five active layers, it reaches a theoretical force of 2.5 N. The force generated by the stack actuators is rather low compared to previously reported stacks prepared from acrylic polymers.^[^ ^54^ ^]^ However, there are applications for DEAs that do not require a large output force, e.g. optical lenses and other optical devices.^[^ ^55^, ^56^, ^57^ ^]^

## 4 Conclusion

We have demonstrated a facile synthesis of a high‐permittivity elastomer with intrinsic self‐healing and thermoreversible properties, which open new possibilities for the fabrication of DEAs. These elastomers' mechanical and dielectric properties can be easily tuned by cross‐linker concentration and polar group ratio variation. Elastomers with 100% polar groups showed a high permittivity of 18.1. The best mechanical properties were achieved for elastomers with 50% polar groups, which possess an attractively high dielectric permittivity value of 12.7. We have proven that the elastomers reversibly soften at temperatures below 110 °C and can self‐heal after damage upon heating to 70 °C. Single‐layer actuators showed lateral strains of 3.8% at a low electric field of 5.2 V µm^–1^ and can self‐repair after an electric breakdown. Moreover, we have demonstrated the recycling of used actuator membranes, after which the initial mechanical properties were restored and new actuators could be fabricated. Combining self‐healing, self‐repairing, and recyclability in one material allows for overcoming different failure modes observed in DEAs. Finally, we have prepared stack actuators reaching actuation strains up to 5.4 ± 0.2% at a low electric field of 3.2 V µm^–1^. To the best of our knowledge, these are the first stack actuators of self‐healable high‐permittivity polysiloxanes ever reported. The low electric field needed for actuation makes these materials attractive for applications in soft robotics. After further reducing the thickness and, therefore, the voltage needed for actuation, the actuators will also be promising as artificial muscles for biomedical applications or optical lenses. Future work will focus on printing these materials in prototype devices.

## 5 Experimental Section

### Materials and Characterization

Unless otherwise stated, all chemicals were reagent grade and used without further purification. Karstedt's catalyst (platinum(0)‐1,3‐divinyl‐1,1,3,3‐tetramethyldisiloxane complex solution in xylene, Pt ≈2%), 2,4,6,8‐tetramethylcyclotetrasiloxane (D_4_H_4_), heptamethylcyclotetrasiloxane (D_4_H_1_), octamethylcyclotetrasiloxane (D_4_), and trivinylmethylsilane were purchased from ABCR. Allyl cyanide, TBPH (solution, 40 wt % in water), and anhydrous tetrahydrofuran were purchased from Aldrich. Tetrahydrofuran was purchased from VWR. More information about characterizations can be found in the Supporting Information. Monomer D_4_^CN^ and cross‐linker tris‐D_4_ were prepared according to the literature.^[^ ^20^, ^58^ ^]^

### Synthesis of the Elastomers

D_4_^CN^ (2.0 g, 3.93 mmol) and the respective amount of initiator TBPH solution (25 µL, 0.92 mol%) were added into a 50 mL three‐necked flask equipped with a magnetic stirrer and dried in HV at RT for 30 min. Afterward, the solution was stirred at 110 °C for 10 min. The respective amount of cross‐linker was dissolved in dry THF (1.0 mL) and added to the reaction mixture (Table 1). The resulting solution was stirred at 110 °C for 20 min under reflux. Afterward, THF was removed in vacuo and the product was molded into one piece on the heating plate to yield a brownish elastic solid. For the elastomers with 50% dimethylsiloxane units, half of the amount of D_4_^CN^ (1.0 g, 1.97 mmol) was used and D_4_ (0.6 mL, 1.97 mmol) was added to the reaction mixture directly after removing the water from the initiator solution in HV.

### (Re)processing to Elastomer Thin Films

**Figure 5:**
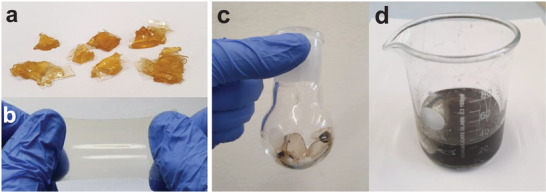
a) Crude elastomer **B1** before processing. b) Film of elastomer **B1** prepared by melt pressing. c) Damaged actuators prepared from elastomer **A4**. d) Solution in THF of depolymerized material **A4** recovered from a damaged actuator before removing carbon black powder by filtration.

Elastomer films were prepared from the crude product (**Figure** **5**a) by melt pressing using a copper spacer with a thickness of 200 µm between two PET sheets coated with PTFE foil. The arrangement was covered with metal plates on the top and bottom side and placed inside a melt press at temperatures between 60 °C and 100 °C. After an equilibration time of 5 min, a pressure of 1000 kg was applied for 1 min. The resulting film was left to cool before removing it from the substrate (Figure 5b).

### Recycling of Actuators

Used actuators from elastomer **A4** (2.0 g) (Figure 5c) were dissolved in dry THF (5.0 mL) at 80 °C under reflux (Figure 5d). The carbon black electrode was removed by filtering the solution through a syringe filter (PTFE, 1.0 µm). After removing THF in vacuo, the reaction mixture was heated to 110 °C and THF (2.0 mL) was added. The solution was stirred for 20 min under reflux before removing THF in vacuo and molding the product in one piece on the heating plate.

## Conflict of Interest

The authors declare no conflict of interest.

## Supporting information

Supporting Information

Supporting Information

Supporting Information

## Data Availability

The data that support the findings of this study are available in the Supporting Information of this article.
