# Supplementary material for: Self‐Healable, Self‐Repairable, and Recyclable Electrically Responsive Artificial Muscles
Source: Adv Sci (Weinh). 2022 Jun 3;9(22):2202153. doi: 10.1002/advs.202202153 (PMC9353453; doi:10.1002/advs.202202153)
Supplement: Supplementary file 1 — Supporting Information [file ADVS-9-2202153-s002.pdf]

ADVANCED SCIENCE
Open Access

Supporting Information

for *Adv. Sci.*, DOI 10.1002/advs.202202153

Self-Healable, Self-Repairable, and Recyclable Electrically Responsive Artificial Muscles

*Johannes von Szczepanski, Patrick M. Danner and Dorina M. Opris**

Supporting Information

Self-Healing, High-Permittivity Polysiloxanes for Recyclable Dielectric Elastomer Actuators

*Johannes von Szczepanski, Patrick M. Danner, and Dorina M. Opris**

J. von Szczepanski, P. M. Danner, D. M. Opris
Laboratory for Functional Polymers
Swiss Federal Laboratories for Materials Science and Technology Empa
Ueberlandstr. 129, 8600 Dübendorf, Switzerland

E-mail: dorina.opris@empa.ch

J. von Szczepanski, P. M. Danner,
Department of Materials
ETH Zurich
Vladimir-Prelog-Weg 5, 8093 Zurich, Switzerland

Keywords: high-permittivity elastomers, dielectric elastomers, soft actuators, self-healing, recycling, soft robotics

Experimental Section

^{1}H-NMR spectra were recorded at 298 K on a *Bruker* Avance 400 MHz NMR spectrometer. Chemical shifts (δ) are given in ppm relative to $CHCl_3$ (δ = 7.26 ppm). IR spectra were recorded in the range of 4000–600 cm^{-1} on a *Bruker* Tensor 27 FT-IR spectrometer equipped with an ATR setup. Tensile tests were performed on a *Zwick* Z010 tensile test machine at a crosshead speed of 500 mm min^{-1} with a pre-load of 2 g. Test specimens with a gauge width of 2 mm and a gauge length of 18 mm were prepared by die-cutting. The stress-strain curves were averaged from at least three independent measurements. Impedance measurements were performed in a frequency range of 0.1 Hz – 1 MHz with a *Novocontrol Technologies* Alpha-A Frequency Analyzer. The root mean square voltage of the probing AC electric signal applied to the sample was 1 V. Dynamic mechanical analysis was carried out on a RSA 3 DMA from *TA Instruments*. Stripes with a width of 10 mm and a length of 25 mm were measured under a

dynamic load of 2 g at 1% strain in the frequency range of 0.05–10 Hz. Rheology measurements were carried out in parallel plate geometry with a Physica MCR301 rheometer from *Anton Paar* with disposable Al stubs (Ø 25 mm) screwed to the rheometer shaft. The gap between the disposable stubs and the cup holder was set to 1 mm. The normal force from the rheometer shaft was maintained at 0 N. The experiments for thermoreversible softening were performed with a heating and cooling rate of 3 K min^{-1}, a constant angular frequency of 1 rad s^{-1}, and a constant deformation of 0.1%. Differential scanning calorimetry was performed on a *Perkin Elmer* double-furnace DSC 8000 with a heating rate of 20 K min^{-1}. Thermogravimetric analysis was performed with a *Netzsch* TG 209-F1 with vacuum-tight thermo-microbalance. Samples were measured in an Al_2O_3 crucible at a heating rate of 20 K min^{-1}. Electromechanical tests were performed with circular membrane actuators. The polymer films were fixed between two circular frames after applying a biaxial pre-stretch of 14.3%. Carbon black powder was applied on each side of the film with a brush as circular electrodes. The electrodes were connected to a FUG HCL-35-12500 HV power supply with aluminum foil. The actuation strain was determined optically with a digital camera detecting the edge between the black electrode and the light silicone film, thus measuring the extension of the electrode diameter. The extension was measured at two different positions and the values were averaged, unless one of the measurements was impaired by local defects of the electrode structure. Stack actuators were prepared by cutting a melt pressed film in quadratic pieces with a length of 1.5 cm. A PDMS matrix filled with graphite nanoplatelets was used as electrode material in the form of cross-linked films. The electrode was cut into rectangular pieces with a shape of 1.0×1.5 cm. The electrode and the dielectric film were manually stacked on top of each other, starting and finishing with the electrode layer. A small piece of the electrode was left jutting out from one side of the dielectric film alternating on two opposite sides, thus placing the third electrode at the same position as the first one and the fourth at the same position as the second one. The electrodes were merged on both sides and connected to the HV source with aluminum foil. The stack actuators were tested with the same voltage source as the single-layer actuators at a frequency of 25 mHz. The change in the thickness of the stack was followed by a laser measuring the distance from the laser to the top of the stack.

Synthesis of Monomer D_4^{CN}*:* In a dried 250 mL 3-necked flask equipped with a reflux condenser and magnetic stirrer, 2,4,6,8-tetramethylcyclotetrasiloxane (D_4H_4) (10.0 mL, 41 mmol) was dissolved in dry toluene (25 mL) under argon. Allyl cyanide (23.1 mL, 287 mmol) and Karstedt's catalyst (60 µL solution in xylene, 2 wt % Pt) were added via a

syringe. The solution was heated to 110 °C and stirred under reflux for 5 days. The reaction progress is followed by FT-IR spectroscopy. After completion, the solvent is removed in vacuo and the product is purified by silica gel filtration (heptane/EtOAc 1:1). The solvents are removed and the product is dried in a high vacuum (HV) at 50 °C for 2 h to yield 18.8 g (90.0%) of viscous, yellowish liquid. ^{1}H NMR (400 MHz, $CDCl_3$, δ): 2.52–2.19 (m, 7H, CH_2-CN), 1.83–1.64 (m, 6H, $\underline{CH_2}$-CH_2-CN), 1.22–1.01 (m, 2H, Markovnikov product), 0.85–0.66 (m, 6H, Si-CH_2), 0.29–0.10 ppm (m, 12H, Si-CH_3); FT-IR: ν = 2960 (CH), 2885 (CH), 2247 (CN), 1458, 1425, 1346, 1261 (s, $SiCH_3$), 1180, 1063 (s, SiOSi), 1039 (s, SiOSi), 866, 787 (s, $SiCH_3$), 746 (s, $SiCH_3$), 696 cm^{-1}.

Synthesis of Cross-linker tris-D$_4$: In a dried 50 mL Schlenk flask equipped with a magnetic stirrer, trivinylmethylsilane (0.97 mL, 6.0 mmol) was dissolved in dry toluene (25 mL). Heptamethylcyclotetrasiloxane (5.29 mL, 18.0 mmol) and Karstedt's catalyst (50 µL solution in xylene, 2 wt % Pt) were added via a syringe and the solution was stirred at RT for 39 h. The reaction progress was followed by FT-IR spectroscopy. After completion, the solvent was removed in vacuo. The product was purified by silica gel filtration (heptane/EtOAc 50:1). The solvents were removed and the product was dried in HV for 2 h to yield 4.14 g (71.1%) of pale brown viscous liquid. ^{1}H NMR (400 MHz, $CDCl_3$, δ): 1.10–0.83 (m, 2H, Markovnikov product), 0.64–0.23 (m, 12H, Si-CH_2-CH_2-Si), 0.22–0.03 (m, 66H, O-Si-CH_3), 0 – -0.1 ppm (s, 2H, $(CH_2)_3$-Si-$\underline{CH_3}$); FT-IR: ν = 2960 (CH), 2908 (CH), 1494, 1259 (s, $SiCH_3$), 1055 (s, SiOSi), 800 (s, $SiCH_3$), 727 (s, $SiCH_3$), 692 cm^{-1} (s, $SiCH_3$).

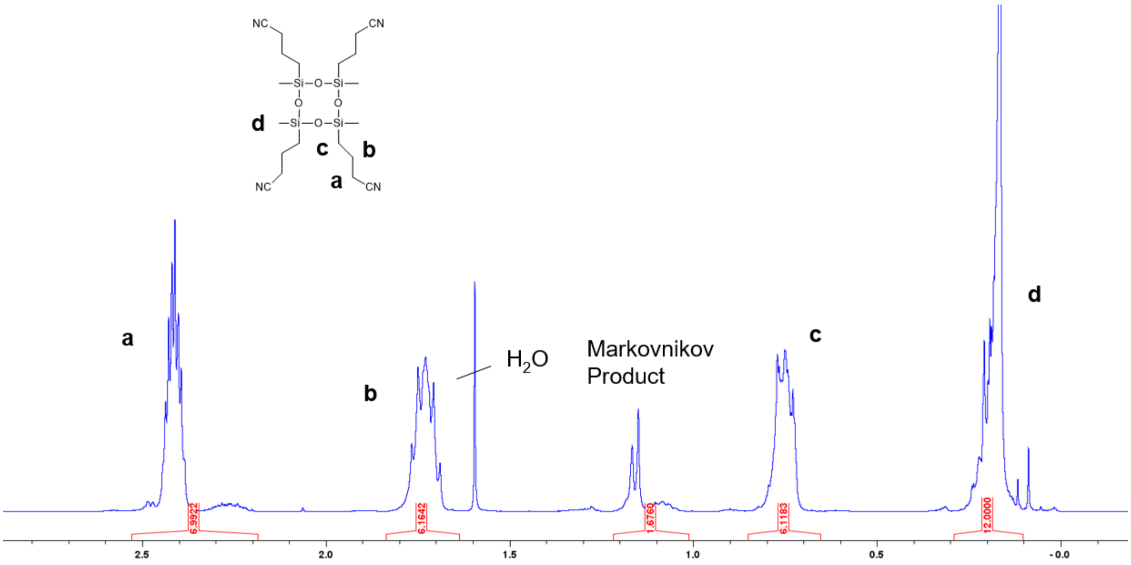

Figure S1. ^{1}H NMR spectrum of monomer D$_4^{CN}$ recorded at 298 K on a Bruker Avance 400 MHz NMR spectrometer.

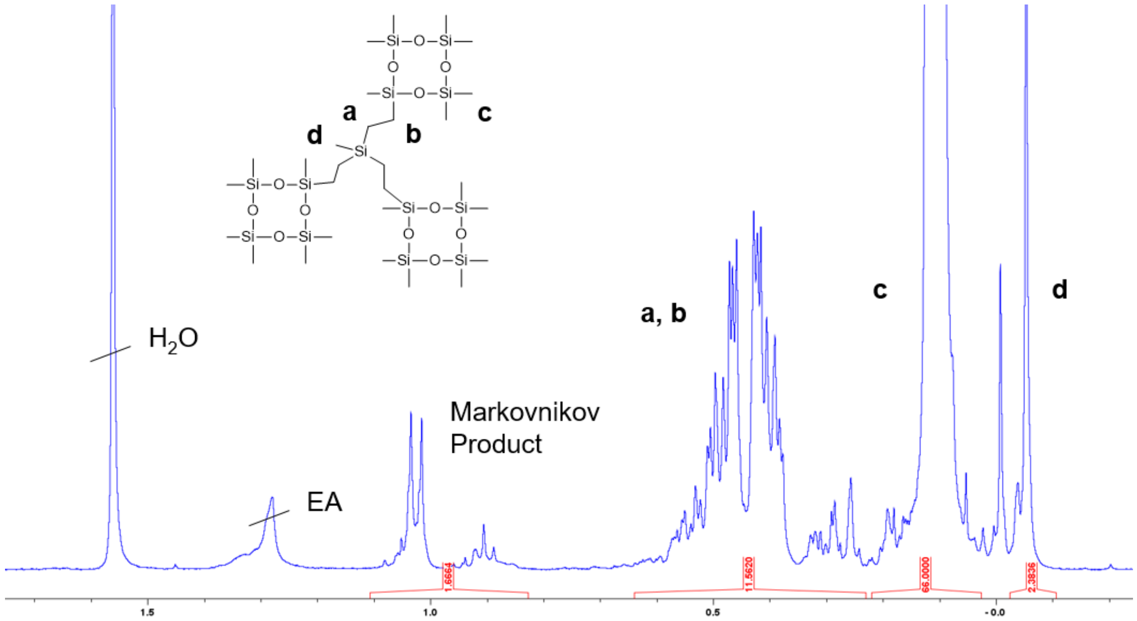

Figure S2. ^{1}H NMR spectrum of cross-linker tris-D_4 recorded at 298 K on a Bruker Avance 400 MHz NMR spectrometer.

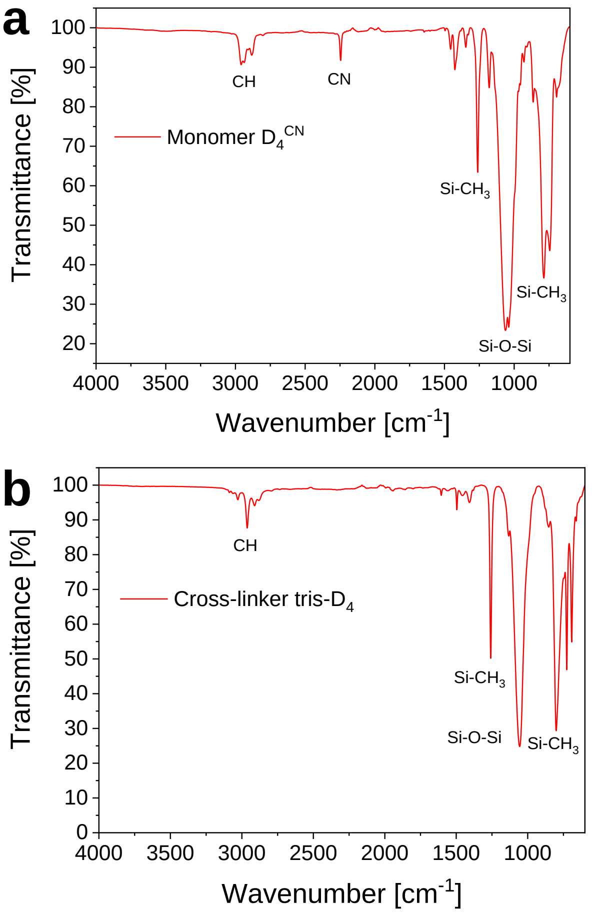

Figure S3. IR spectra of monomer D_4^{CN} (a) and cross-linker tris-D_4 (b) were recorded on a Bruker Tensor 27 FT-IR spectrometer equipped with an ATR setup.

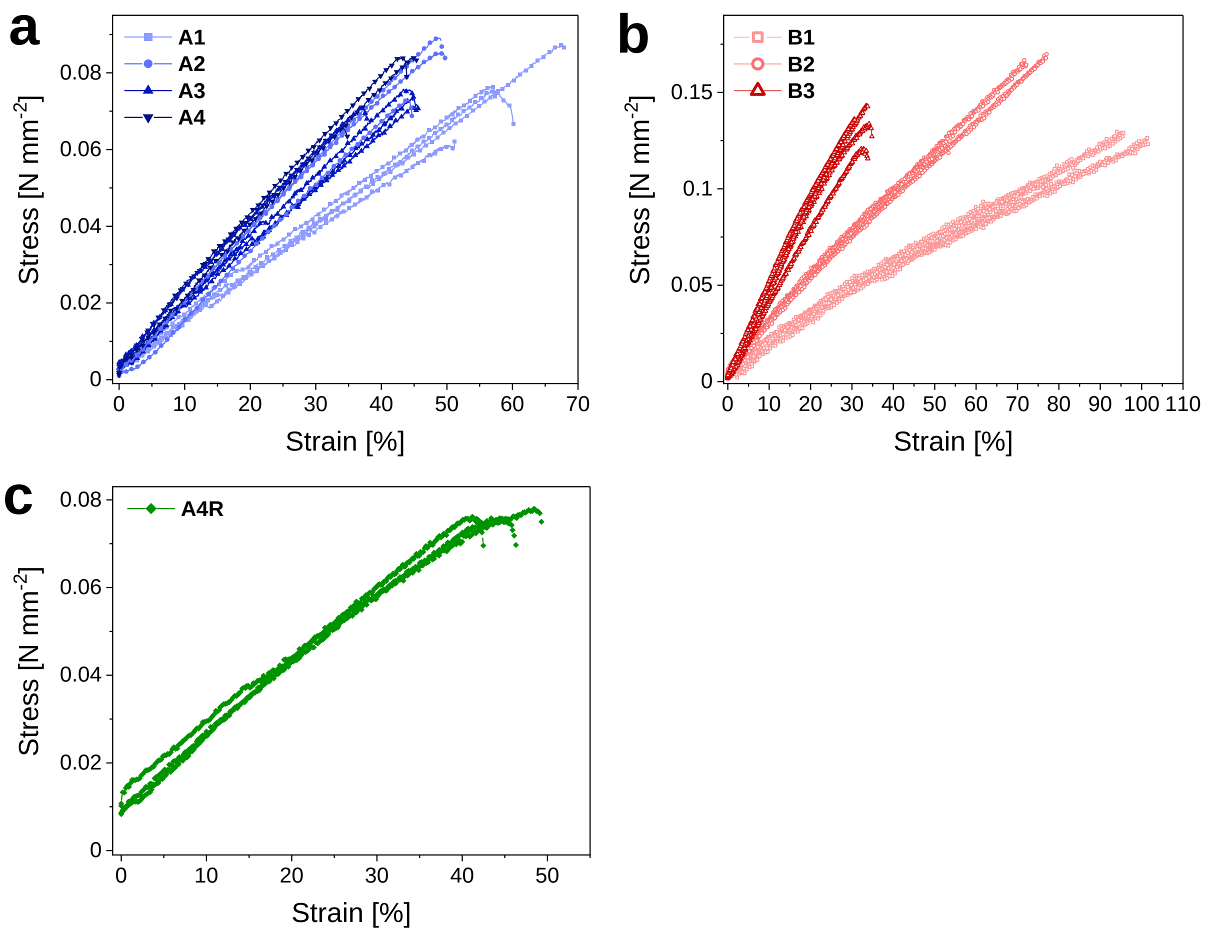

Figure S4. Tensile test curves of elastomers **A1-A4** (a), **B1-B3** (b), and **A4R** (c) were performed on a Zwick Z010 tensile test machine at a crosshead speed of 500 mm min^{-1} with a pre-load of 2 g. Test specimens with a gauge width of 2 mm and a gauge length of 18 mm were prepared by die-cutting.

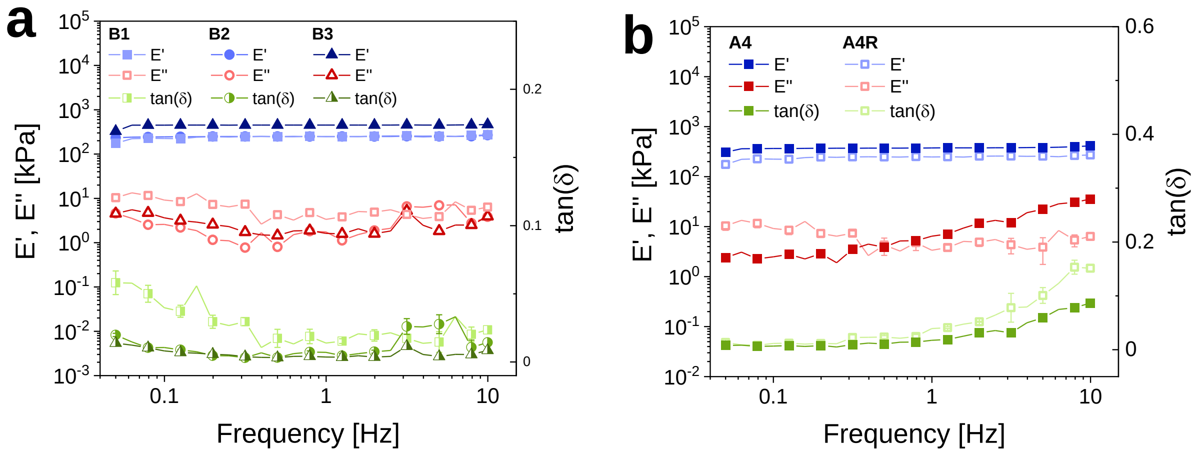

Figure S5. DMA curves of elastomers **B1-B3** (a) and **A4** and recycled elastomer **A4R** (b). DMA was carried out on a RSA 3 DMA from TA Instruments. Stripes with a width of 10 mm and a length of 25 mm were measured under a dynamic load of 2 g at 1% strain in the frequency range of 0.05–10 Hz. If not shown, error bars are smaller than the symbol size.

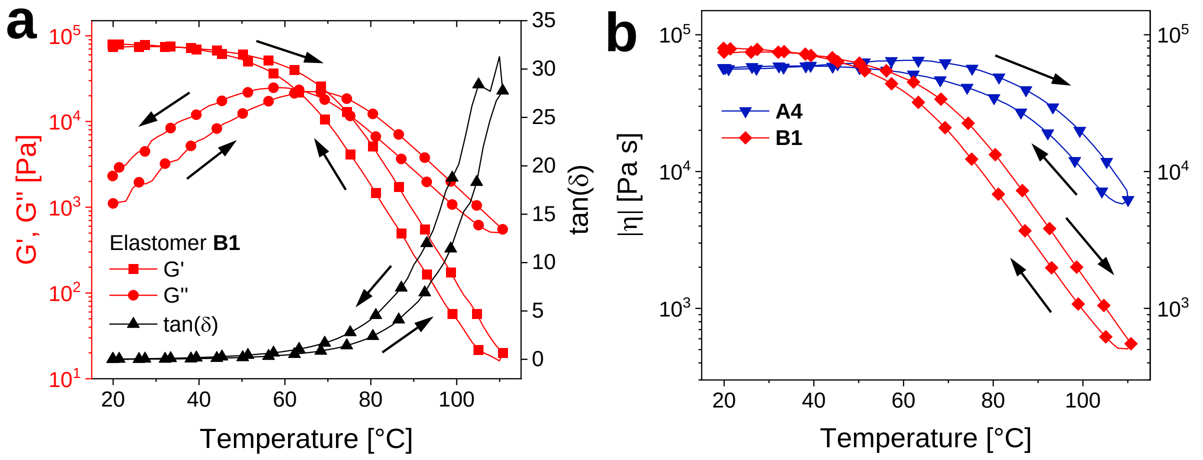

Figure S6. Temperature-dependent moduli of elastomer **B1** (a) and temperature-dependent viscosity of elastomers **A4** and **B1** (b). Rheology measurements were carried out in parallel plate geometry with a Physica MCR301 rheometer from Anton Paar with disposable Al stubs (Ø 25 mm) screwed to the rheometer shaft. The gap between the disposable stubs and the cup holder was set to 1 mm. The normal force from the rheometer shaft was maintained at 0 N. The experiments for thermoreversible softening were performed with a heating and cooling rate of 3 K min^{-1}, a constant angular frequency of 1 rad s^{-1} and a constant deformation of 0.1%.

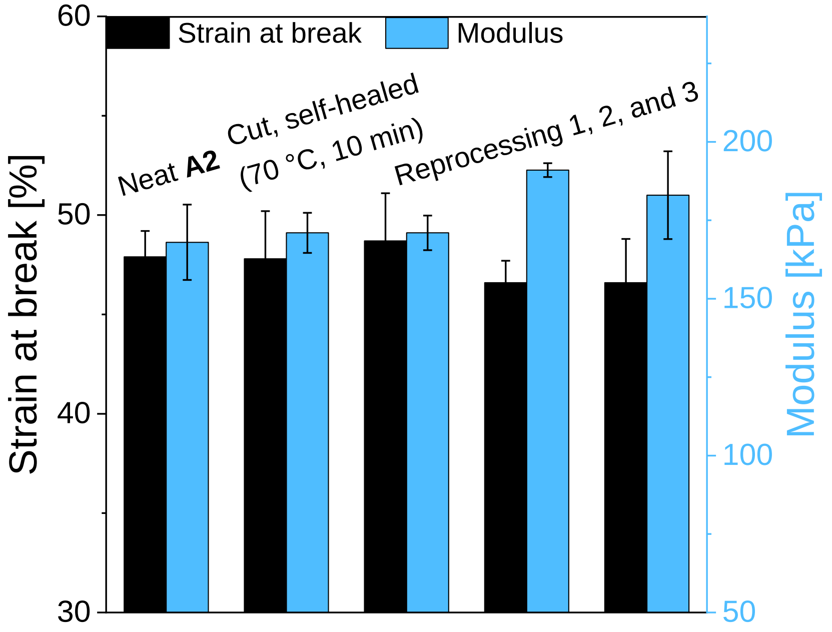

Figure S7. Self-healing experiments of elastomer **A2**. The initial mechanical properties are restored after cutting and heating to 70 °C for 10 min. Also, by up to three processing cycles the mechanical properties are only slightly impaired.

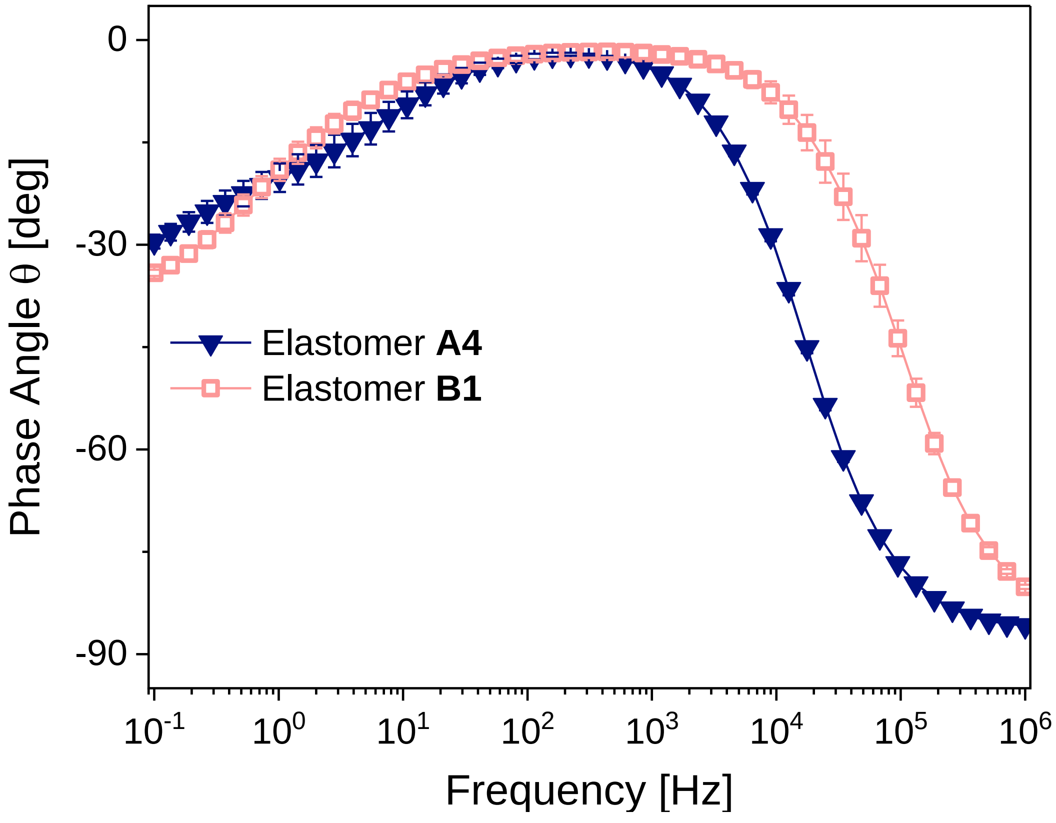

Figure S8. Phase angle between voltage and current as a function of frequency extracted from the impedance spectroscopy measurement of the elastomers **A4** and **B1**.

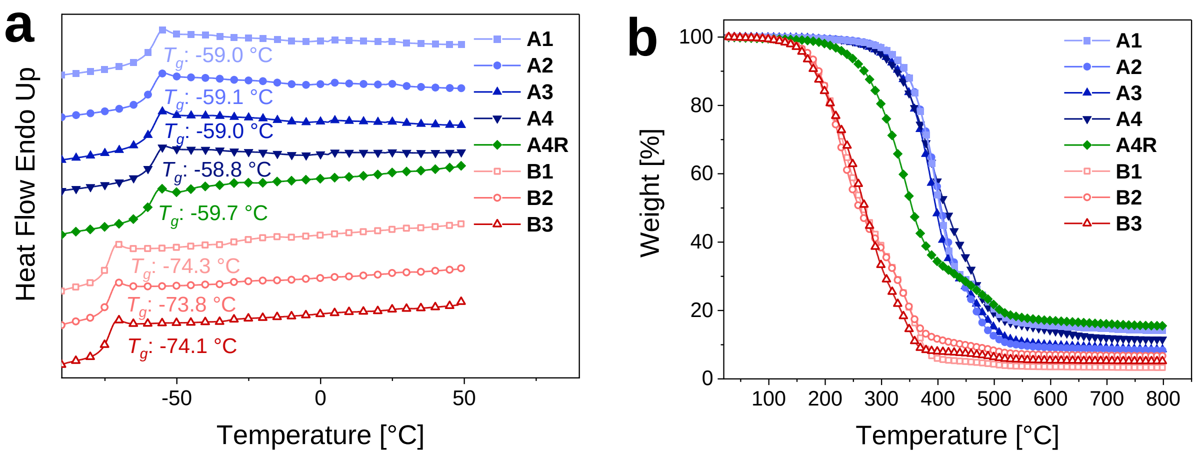

Figure S9. DSC curves (a) and TGA curves (b) of all elastomers. DSC was performed on a Perkin Elmer double-furnace DSC 8000 with a heating rate of 20 K min^{-1}. TGA was performed with a Netzsch TG 209-F1 with vacuum-tight thermo-microbalance. Samples were measured in an Al_2O_3 crucible at a heating rate of 20 K min^{-1}.

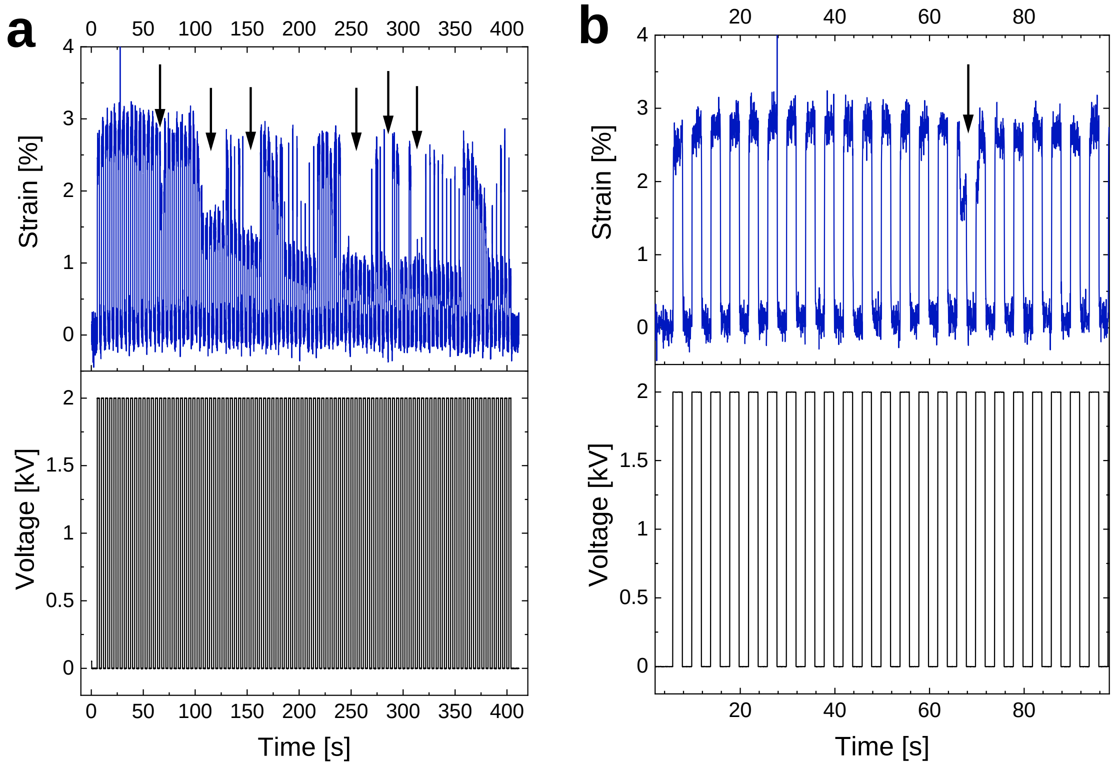

Figure S10. Self-repairing of an actuator prepared from a membrane of elastomer **A4** with a thickness of 288 µm. Overview of 100 cycles at 2000 V (6.9 V µm^{-1}) (a) and enlarged section of the first 23 cycles (b). Breakdown events are indicated with arrows.

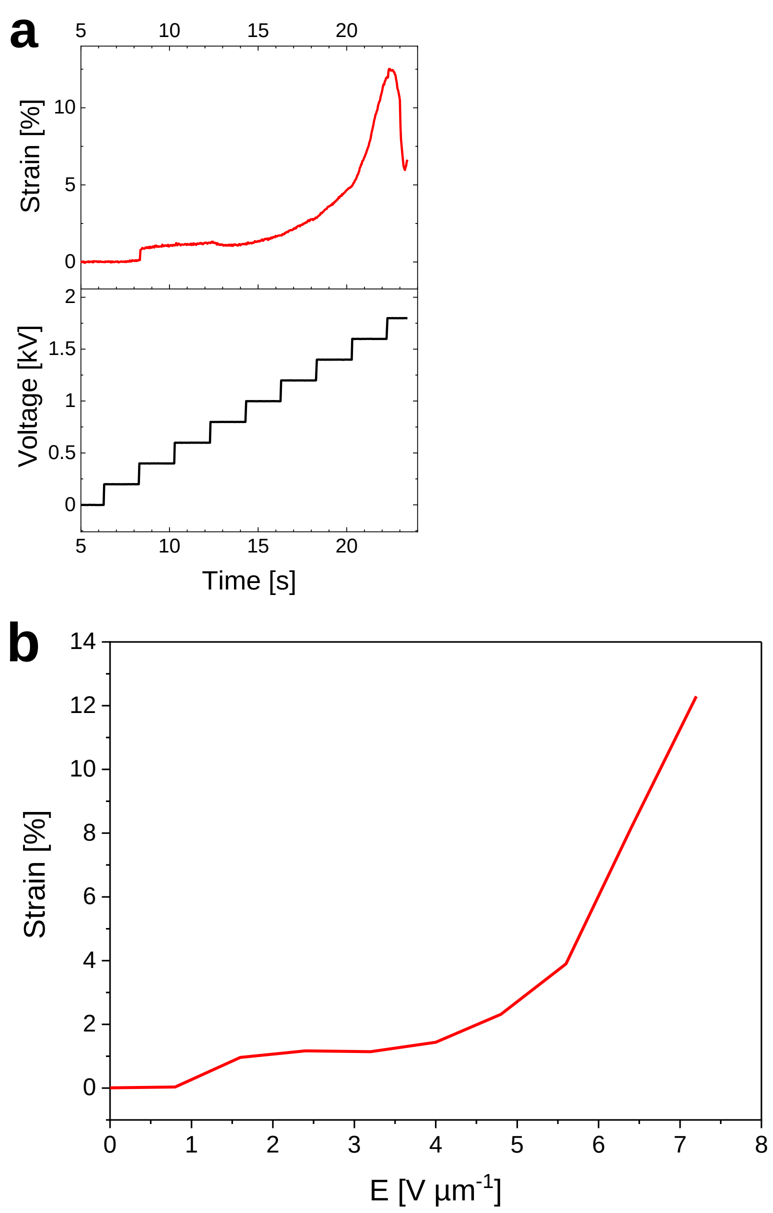

Figure S11. Step increase of an actuator prepared from a membrane of elastomer **B1** with a thickness of 250 µm. The actuator shows a large lateral strain of 12.3% at 7.2 V μm^{-1}. Strain and voltage are plotted as a function of time (a) and strain is plotted as a function of the electric field (b).

Videos:

S1: Stack actuator prepared from elastomer **A4** 8 times speed
S2: Stack actuator prepared from elastomer **B1** 8 times speed
